# Social Circus for People with Disabilities: A Video Analysis through the Lens of the MOHO

**DOI:** 10.1155/2021/6628482

**Published:** 2021-03-09

**Authors:** Bianca A. D. Thompson, Kieran Broome

**Affiliations:** ^1^School of Health & Sport Sciences, University of the Sunshine Coast, Maroochydore, QLD 4558, Australia; ^2^Research Services, Good to Better, Imbil, QLD 4570, Australia

## Abstract

**Background:**

Social circus is a branch of circus that primarily focuses on personal and community development, rather than an elite level of professional artistry required of traditional circus. Social circus engages participants in circus activities such as juggling and acrobatics with therapeutic aims such as building confidence or developing life skills. While there is a growing body of literature around social circus, there is currently limited literature exploring the interface between social circus and occupational therapy theory.

**Objective:**

This study is aimed at examining existing examples of social circus for people with disability (via YouTube videos) through the lens of the Model of Human Occupation (MOHO) to consider the link between social circus and contemporary occupational therapy practice.

**Methods:**

The study utilised video analysis as the guiding methodology. A two-part qualitative thematic analysis was conducted on transcripts of YouTube video audio and on-screen text, as well as visual analysis of the corresponding imagery.

**Results:**

Social circus provides people with disabilities opportunities to actively participate and experience dignity of risk, independence, and autonomy, in a safe and inclusive environment amongst others. As a highly flexible activity (in structure, timing, tasks, outcomes, and environments), social circus accommodated differences in capacities and provided opportunity for the development of skills, both circus-specific and generalisable to everyday life. Social circus allowed people with disability to shape new identities as performers, friends, and members of a community.

**Conclusion:**

Social circus offers a unique means for successfully attaining and achieving a wide range of occupational outcomes for people with and without disability across a diverse range of settings. Utilising an occupational therapy lens led to insights around the social circus environments, development of identity and transference of circus skills to everyday tasks and occupations, that were not previously acknowledged in the social circus literature. Our findings support social circus implementation and collaboration within contemporary occupational therapy practice.

## 1. Introduction

All people with disabilities deserve the right to participate in social, physical, and community-based activities as such activities are beneficial for one's overall health, self-improvement, social skills, and wellbeing [1]. While there is a growing body of literature around social circus [2–18], there is currently limited literature exploring the interface between social circus and occupational therapy theory. In this paper, we will be exploring the experiences of social circus for people with disabilities through the Model of Human Occupation (MOHO) [19] to consider the link between social circus and contemporary occupational therapy practice.

Since its emergence in the early 1990s, social circus has been met with ever increasing acceptance and has become a pioneering tool for social intervention globally [2]. Social circus is a branch of circus that primarily focuses on personal and community development, rather than an elite level of professional artistry required of traditional circus. Defined as an all-encompassing art form, sport, and/or leisure activity, social circus remains adaptable to a range of participants and situations while endeavouring to offer a range of circus techniques, which may include trampolining, stilts, unicycling, juggling, and acrobatics [20]. Social circus is further based on seven core principles: “creation of a safe and fun space”; “expression, creation, and performance”; “links with the community”; “collaboration between social intervention and circus”; “duration over time and continuity”; “partnerships”; and “a participant-centred process” [2]. Accordingly, initiatives have, and continue to target, a broad spectrum of individuals living with and experiencing various complexities (e.g., individuals experiencing mental health difficulties [8–10], young adults living with physical disabilities [7], people with vision impairment [11], young people with autism spectrum disorder [12], older adults [11], at-risk youth [13], and individuals living in marginalised communities [14] and socially volatile areas [15]).

People with disabilities participate less frequently in social activities, skill-based activities, physical activities, and self-improvement activities than people without disabilities [21–24]. Furthermore, the activities that they participate in are most commonly completed at home, with family members, rather than with friends, or the wider community [25, 26]. Social circus is proposed as an intervention to facilitate occupational engagement and participation, as circus is a multifaceted art form that is adaptable to a range of participants and situations. Social circus is quintessentially a branch of circus that “encourages the development of self-esteem and the acquisition of social skills, artistic expression and occupational integration” (p.14 of [2]). At present, many therapeutic benefits are posited in the literature [3–6], with research further supporting its utilisation within occupational therapy practice and the wider health sphere [3, 7].

To date, the efficacy of social circus is emerging with a wide range of different studies conducted internationally, with academics and artists defining social circus as a progressive intervention that provides opportunities for physical, cognitive, and psychosocial benefits. Reg Bolton, a pioneer of social circus, asserted that in circus, you achieve the impossible, defining circus as a medium to explore risk, experience trust, hard work, aspiration, self-individuation, and fun [16]. Such benefits continue to be widely reported in the literature, with participation noted to positively aid physical and mental well-being; foster social participation and social skills; provide opportunities for individuals to experience joy, trust, creativity, and fun; and promote opportunities for enhanced self-esteem, self-improvement, resilience, and confidence.

Consequently, the evidence would appear to indicate that social circus may be a suitable intervention within occupational therapy practice, given extensive opportunities exist for enablement, adaptation, and participation. While not widely considered, emerging evidence supports social circus collaboration and implementation within occupational therapy practice with Maglio and McKinstry [3] suggesting that when implementing circus programs in collaboration with occupational therapy practice, circus programs should be innately client-centred and structured to meet the individual needs of participants. They propose that occupational therapists and circus trainers who work together through a collaborative process better enable each profession to successfully develop and deliver social circus programs [3]. Their hypothesis has since been supported by Loiselle et al. [7], who assert that occupational therapists remain the health professional of choice in the collaborative implementation and delivery of social circus programs [7].

Despite the clear synergies between occupational therapy and social circus, the overall quality and trustworthiness of findings should be considered as findings have not undergone rigorous analyses. Furthermore, it might be possible to argue that existing studies are typically biased towards the fields and theoretical viewpoints of professions such as public health, education, and psychiatry. Occupational therapy offers unique theoretical lenses. Social circus has not yet been examined through an occupational therapy theoretical lens, such as the Model of Human Occupation [19].

Kielhofner's Model of Human Occupation (MOHO) [19] is arguably the most widely utilised model in occupational therapy research worldwide [27]. The occupation-focused model delineates the dynamic process in which people engage in occupations of interest to achieve a sense of competence and identity [19]. MOHO offers occupational therapists a conceptual framework to explicate how occupations of interest are selected, organised, and undertaken within the environmental context [19].

The MOHO conceptualises these convolutions through the following constructs: *Environment* (the social and physical context that facilitates occupational engagement), *Volition* (motivation for occupation), *Habituation* (routine patterning of occupational behaviour), *Performance capacity* (level of skilled performance), *Participation* (what we do in the broadest sense), *Performance* (discrete acts or units of doing), *Skill* (observable goal directed actions that make up occupational performance), *Occupational Identity* (a sense of who we are and wish to become), *Occupational Competence* (ability to perform occupations with skill and ease), and *Occupational Adaptation* (making needed changes to continue to engage in desired activities or developing new activities) [19].

While no studies have currently used the MOHO to explore and categorise the benefits, contexts, and experiences of social circus, the themes currently identified in the literature align well with the MOHO. For example, when considering Seymour's [12] and R. Taylor and C. Taylor's [17] findings concerning social circus for children, the constructs of occupational competence, skill, and performance are commonly identified. Circus skills training provides opportunities for attainment of physical and gross motor skills, enhanced communication, self-esteem, self-confidence, positive changes in behaviour patterns, and opportunities for children to learn social, affective, and cognitive skills, while having fun [12, 17]. The previous literature makes few explicit connections between social circus and occupational therapy theory. The aim of this study is to examine existing examples of social circus for people with disability (via YouTube videos) through the lens of the MOHO.

## 2. Materials and Methods

### 2.1. Ethics

Ethics approval was obtained from the Human Research Ethics Committee of the University of the Sunshine Coast (Ethics approval number OE20027).

### 2.2. Study Design

The study utilised video analysis as the guiding methodology. A two-part qualitative thematic analysis was conducted on transcripts of YouTube video audio and on-screen text, as well as visual analysis of the corresponding imagery.

### 2.3. Search Strategy

Due to the constant turnover of YouTube videos, a YouTube search was conducted and finalised on the 3rd of May 2020. The broad search terms “circus” and “disability” was used. The search term was entered in the YouTube search bar at https://www.youtube.com, and no filters were applied. All identified videos were screened in sets of 10 by the first author to determine relevance. Screening occurred until such time that no further videos were procured that met the inclusion criteria. Throughout the screening process, each video was independently reviewed in a separate internet tab to avoid alterations to the YouTube search.

### 2.4. Inclusion and Exclusion Criteria

Inclusion criteria applied to video selection were (1) the video was publicly available and (2) the video contained humans participating in social circus. Videos were automatically excluded if (1) the video was of very poor audio-visual quality where footage and/or audio content could not be discerned; (2) the video contained the same title or a slightly different title with identical content of an already included video (i.e., the video appeared multiple times)—in this occurrence, the earliest retrieved video was included; and (3) the video was a shortened version of another retrieved video. All further videos, including videos in languages other than English, were included for coding and analysis. In non-English language videos, only visual analysis and analysis of on-screen text and subtitles in the English language were included. It should be noted that when there was any ambiguity regarding a video and its inclusion, discussions occurred between researchers until consensus was reached.

### 2.5. Sample

Given the nature of YouTube, an unidentified number of videos were retrieved from the complete electronic search. Postscreening, a total of 26 purposefully selected YouTube videos were identified. All videos deemed relevant postscreening were procured for subsequent review, transcription, and analysis in NVivo 1.2. YouTube videos were imported via NCapture (a free web-browser extension for Internet Explorer and Chrome), with YouTube videos containing primarily English audio further transcribed with NVivo Transcription.

In total, the sample equated to 2 hours, 39 minutes, and 17 seconds of video footage. Videos ranged in length from 30 seconds to 27 minutes and 4 seconds, with a median of 3 minutes and 26 seconds. All included YouTube videos were uploaded between the 11^th^ of December 2008 and the 9^th^ of November 2019. The majority of videos (92%) were posted within the past ten years; 65% were posted in the last five years. The sample was representative of social circus across multiple continents with footage taken in places including Australia, the United Kingdom, France, Ethiopia, the United States of America, the Middle East, and Myanmar.

### 2.6. Data Analysis

Due to the emerging nature of the field of theoretical video analysis, a novel seven-stage analysis approach was developed as per Table 1, reflecting the processes currently used in video analysis literature [28–30] as well as established analysis processes such as thematic analysis [31].

### 2.7. Specifics of the Theoretical Video Analysis

The video analysis was conducted as per the seven stages of theoretical video analysis, whereby all videos were reviewed, then interpreted and coded through the theoretical lens of the MOHO. Through analysis, both the primary and subconstructs of the MOHO: *Volition* (*interests*, *personal causation*, and *values*); *Habituation* (*habits*, *roles*); *Performance Capacity* (*objective*, *subjective*); *Participation*, *Performance*, and *Skill* (*motor*, *process*, and*communication* and *interaction*); *Occupational Identity*; *Occupational Competence*; *Occupational Adaptation*; and *Environment* (*occupational*, *physical*, and *social*) were considered. Researchers additionally documented disabilities and conditions of social circus participants as well as geographical locations where social circus was conducted as reported by on-screen text, social circus participants, or their significant others. Ages and age ranges (i.e., children, young people, and adults) were also noted in instances where they were reported or it was highly discernible.

One content coder (i.e., the first author) independently analysed each video; the same content coder coded all 26 videos of the sample. Throughout the coding process, all aspects of each video were considered, including coding of video audio (e.g., speech, laughter, and music), visuals (e.g., environments, body language, occupations, and skills), textual visuals (e.g., on-screen text, subtitles, signs, and slogans), YouTube transcripts, and YouTube titles. Each aspect deemed relevant was then contemporaneously matched to a construct of the MOHO, at which time, the YouTube video was paused and coded where applicable. As an example, “juggling” was categorised as per the MOHO construct of *Performance*, whereas “public speaking” was categorised as a *Skill*, specifically a *communication and interaction skill*.

It should be noted that each YouTube video and its corresponding transcription were reviewed until such time that the content coder considered all identified components sufficiently categorised and reported. To further enhance the trustworthiness and confirmability of the coding process, the tool of “critical friend” as per Smith and McGannon [32] was adopted, whereby the second author offered a critique of the coder's work. In total, 398 agreed codes, i.e., where the coder and critical friend agreed on the degree to which the codes accurately reflected the conceptual model, were identified. Codes consequently inferred the underpinning of the themes as reported in the results. Due to the publicly available nature of the data, still images and quotes were extracted and presented to illustrate the categories.

### 2.8. Rigour

A structured approach to analysis was undertaken based on the novel method of theoretical video analysis described above. The structured process, use of NVivo, and maintenance of records of coding, categorisation, and thematic development provided a comprehensive audit trail. While utilisation of an evidence-based conceptual framework was applied throughout the process of analysis, it is acknowledged that parts of the data analysis procedure may have increased subjectivity due to researchers' prior experiences with circus. The first author acknowledges prior experiences with circus through her professional background in dance and the performing arts. The second author has worked as a circus facilitator, including social circus. While the authors' backgrounds in circus as well as professional grounding in occupational therapy may have influenced interpretations, their experiences provided enhanced opportunities for more accurate identification of the various circus performance components and environmental factors which may not have been captured otherwise.

## 3. Results

A range of disabilities and conditions was captured as reported by social circus participants, their significant others, or on-screen text, which included physical, intellectual, cognitive, and learning disabilities: brittle bone disease, Down syndrome, Hallermann-Streiff syndrome, fibromyalgia, anxiety, people with complex health needs, visually impaired or blind, hearing impaired or deaf, amputees, autism, sensory impairments, attention deficit hyperactivity disorder, dyslexia, and learning difficulties. Researchers also noted age ranges of participants which included children, young people, and adults. Such factors highlighted the all-encompassing and inclusive nature of social circus.

### 3.1. MOHO

#### 3.1.1. Volition

Social circus participants were inspired by a range of different motivators. A strong theme across videos was sense of *fun*. Participants were regularly noted to be laughing, smiling, and celebrating their successes. Activities that an outsider may see as intrinsically *risky*, for social circus participants elicited a sense of *joy*. For example, as Ashley noted his first experience with the lyra, “I really enjoyed it. I've never flown before. I was a bit nervous when I first got on, but then I had a big smile on my face.”

**Figure 1:**
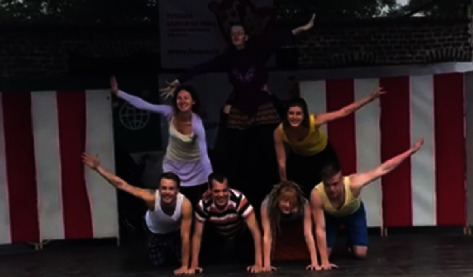
Performers show off and receive applause for their performance [33].

The sense of fun was also coupled with a sense of achievement in *trying something new*. For example, as social circus participant Sam Taylor noted, “It opened my eyes to what I can really do and can really achieve.” This sense of achievement was reinforced by feedback from the audience, as participants were able to show off (see Figure 1).

*Failing* and *falling* were further noted to be an integral part of social circus. As Scot (Ted) Tornaros, circus trainer, reported:

“For people with disabilities, who um, you know, their day to day life is filled with protecting them from falling—protecting them from this. They can really push the limits. They can really get out of their comfort zone. If they take a little spill, it's all in a safe, controlled environment. But, at the same time, it's not wrapping them up in cotton wool and making it so safe that there's no, you know, sense of achievement.”

#### 3.1.2. Habituation

Social circus participation occurred at *flexible times*, with participation most frequently reported to occur on a weekly basis. Such habits consequently provided opportunities for repetition and practice. Sam Taylor, social circus participant noted, “… adding a new type of exercise to my weekly routine. I thought it was really important to try new things and keep you more active and fit through the use of silk.”

Participants were also provided the opportunity to experience *autonomy*. Veronica Astar, service manager, noted:

"One of the things we wanted people to do was to travel here independently. So that was part of the remit of the group, which everybody did. So, it was around people having a sense of independence and then coming together at a certain time on a certain day. And that's worked really well."

Within the social circus sphere, *embracing roles* which included students, participants, volunteers, and duo partners were identified. Interestingly, participants reported that through circus, they *could be a different person*; this was exemplified by the use of costumes and masks where people could personify a character and identity other than their own. Participants were likewise provided opportunities to become storytellers, thus sharing the realm of theatre, imagination, and creativity with their peers and audience members.

#### 3.1.3. Performance Capacity

**Figure 2:**
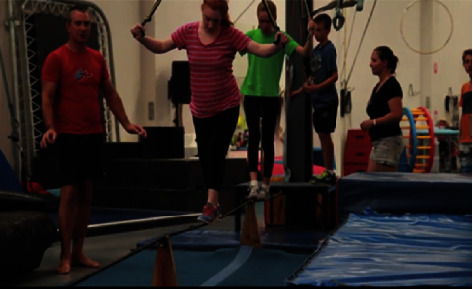
Social circus participants learn to tightrope walk [34].

Due to the physical and creative nature of the art form, an extensive breadth of performance capacities was identified. Highly *transferable capacities* relevant to everyday tasks and occupations included components such as balancing, grasping, reaching, strength, and posture (see Figure 2). Such components remained innately intertwined within the various circus tasks and skills, with competence and practice enhancing participation.

*Teamwork* was identified as an important component of participation. As circus facilitator Kate Priddle noted:

“Circus is about being a team. It's about trust, about working together. It's about finding people's uniqueness and celebrating that whatever it might be right through to, you know, the sideshow days of really celebrating people's differences. So that's sort of the ethos of circus. And that's what we love about it, that it's non-competitive and that it really is inclusive. Essentially at its core. So, it was, it's a perfect platform to extend that and open that up to people of all abilities.”

#### 3.1.4. Participation

Through social circus, participants were provided extensive opportunities to participate. While the key opportunity provided for *participation* was inclusion and engagement in a physical art form, various other opportunities were eventuated as a result of involvement. Commonly identified participation experiences included participation in friendships, educational experiences, and live performance opportunities. However, the most significant experience identified was that through social circus, people with disability are provided the opportunity to *actively participate*.

#### 3.1.5. Performance

**Figure 3:**
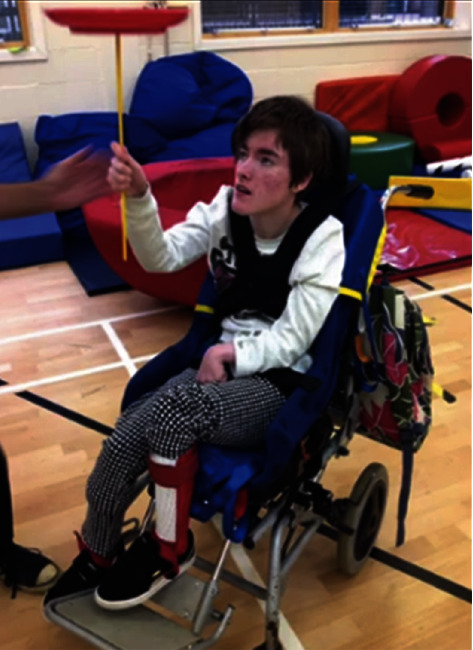
Participant focusing and spinning plates independently [35].

A strong premise identified was the *variety within the circus*, particularly the contrast between social circus and contemporary circus. Contemporary circus performance components such as the Wheel of Death, Aerial acts, Russian bar, Cyr wheel, and Acrobatics were identified. Performance components not typically associated with contemporary circus such as chilling out, balancing peacock feathers, playing games, climbing stairs, jumping, spinning plates (see Figure 3), stretching, and packing up were more so eminent within the social circus sphere.

#### 3.1.6. Skill

Social circus participants utilised an array of motor, process, communication, and interaction skills through their engagement with circus. Communication and interaction skills were enhanced through the person's *patience and respect for others*. Participants were regularly noted to be listening to others, taking turns, and sharing. Opportunities for communication were enriched through both *verbal and non-verbal communications and interactions*. As one person noted:

“We play games that helps to create a really good social environment where the children can join in. They can listen to each other; they can share things; they can take turns. But, it also encourages people to think really creatively.” (Video narrator)

Process skills such as following instructions, attention, copying, watching, and learning were all so noted. Coordination, bilateral integration, hand-eye coordination, and visual attention featured across the majority of circus tasks.

**Figure 4:**
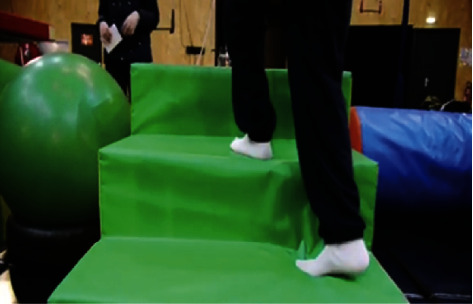
Participant climbing stairs as part of a circus obstacle course [36].

Motor skills appeared only limited by creativity and choreographic licence. Participants were frequently jumping, bending, catching, and linking arms, amongst other skills. Circus allowed people with disabilities to showcase their motor abilities without focusing on their *dis*abilities. Interestingly *everyday activities* such as climbing stairs (see Figure 4) and tying shoelaces were further included, which exemplified the *transference of circus skills to everyday tasks and occupations*.

#### 3.1.7. Occupational Identity

The social circus experience is about *being and becoming*. Through social circus, persons can craft their identity through the vast opportunities that exist for enablement, adaptation, and participation. Persons are likewise provided opportunities to learn, embrace, fail, and succeed, thus challenging themselves to choose what they can be and who they can become. For example, a young man relates his experiences of social circus to the song “Go the Distance” from the Disney classic “Hercules.”

#### 3.1.8. Occupational Competence

**Figure 5:**
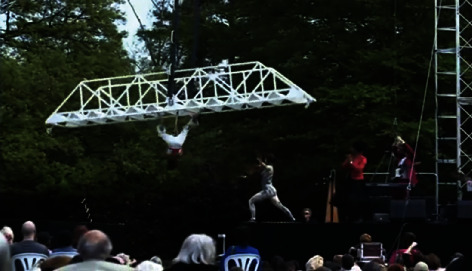
Disabled performers reclaiming the term “freak show” [37].

Children, young people, and adults of a range of competence levels and abilities participated in social circus across an array of settings; in essence, social circus provided *a safe space for people of all abilities*. Additionally, while small displays consisting of novices occurred at community halls, highly skilled performers delivered extraordinary choreographic acts live on stage (see Figure 5). Such *breadth of competence* not only captured but reiterates the adaptive and inclusive nature of social circus.

#### 3.1.9. Occupational Adaptation

A wide range of *physical and mental benefits* was reported by participants and their support networks. Improved mobility, strength, coordination, and balance were frequently reported. Cassia Moore, a social circus participant, noted, “Basically, I feel myself getting stronger, amazing!” Similarly, Sam Taylor, a social circus participant, reported, “I feel more mobile, and I feel more active, and I feel the way I can move around a bit more.”

Participants and their support networks additionally noted that the circus sphere provides opportunity to *express who you are and gain confidence*. As one mother described her daughter's experience with social circus, “What a special experience … the confidence building is huge” (Circus Mum).

*Personal Growth* was innately entwined with the circus experience, with individuals frequently reporting that through circus they were able to get out of their comfort zone and overcome boundaries and challenges. Kyle Burns, social circus participant reported, “I have learned to be more confident, because before I started coming here, I suffer [*sic*] from quite severe stage fright. Now, I've been doing this with all the other actors watching me, it's been helping me conquer that.”

Such factors allowed opportunities for participants to experience *trust* (*with self and others*), *creation*, *independence*, *talent*, and *achievement*. As one circus facilitator summarised her groups social circus experience, “It's lovely to see all those flow on effects can be of, of achieving of feeling, an independence, of feeling an engagement, of spending time with peers, meeting new people” (Kate Priddle, Circus facilitator).

#### 3.1.10. Environment

*Diversity* was the overarching theme within the occupational environment with persons from underprivileged neighbourhoods partaking through to persons at the European Championships and London Olympics. *Diversity* was evident amongst the wide range of companies, inclusive organisations, and schools currently engaging with social circus internationally.

“Circus is such a rich and diverse, culturally diverse community as it is. There's, it's so natural for it to be integrated with different ability levels, and I think that sense of community is what makes it more open and draws people with disability in.” (Debbie Roach, Circus performer)

**Figure 6:**
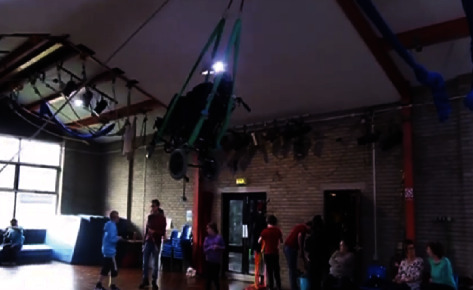
Participant in wheelchair suspended from the roof [35].

Social circus participation was noted to occur in a wide range of *highly adaptable and accessible spaces and places*. Locations most commonly included outdoor spaces, stages, community halls, studios, or stadiums. Such spaces remained highly adaptable and accessible to participants as various props, surfaces, sounds, and lighting requirements were tailored towards individual or organisational needs (see Figure 6).

Within the social environment, *inclusion* was evident as *people could express themselves through a range of communicative forms*. Various opportunities existed for both verbal and nonverbal communication. Communication in the form of sign language and audio description of social circus performances not only enhanced participants' experiences but provided *increased cultural and creative opportunities* for members of the wider community who chose to engage. Community integration and the noncompetitive nature of the circus spaces also signified *inclusion*. As social circus trainer Scot (Ted) Tornaros noted, “It makes people realise that it's not just for the flexible gymnasts. It's not just for the tough, tough guys. It's for, it's for everyone.”

**Figure 7:**
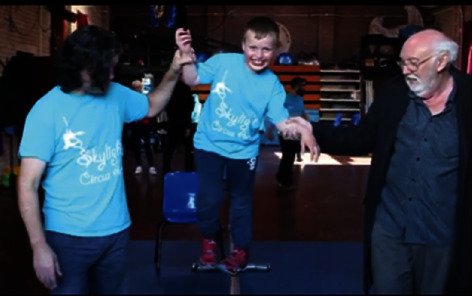
Participant receives a helping hand at a social circus class [35].

People were frequently seen socialising, encouraging, and inspiring each other, which transpired to persons *meeting new people* from a variety of backgrounds. Various social supports of participants such as family, friends, and community members were commonly in attendance at classes, rehearsals, and performances which offered additional *social support* to participants when required (see Figure 7).

Through *inclusion*, opportunities for social interaction were provided, which offered the opportunity for *connection*. *Connection* was evident in the form of teamwork where people were frequently helping others (i.e., acting as spotters) or participating in required partnerships (i.e., duo partners) of the circus sphere. Such occurrences were reported to result in people building relationships and establishing bonds. Social encounters were reported to translate into *friendships outside the circus environment*:

“I think one of the most positive things that's come out of the whole project is the sense of friendship that's developed between the group … it was truly, a really nice thing to see, and it has developed even now, so we have got people within the group, not just meeting together when they come here on a Thursday afternoon, but meeting each other outside of the group, texting one another, telephoning each other.” (Veronica Astar, Service manager)

**Figure 8:**
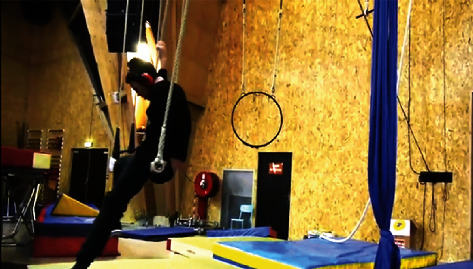
Participant swinging on the trapeze [36].

Interestingly, despite existing evidence, social circus on occasion was *not always social*. A considerable number of videos contained individuals practising independently or parallel to others (see Figure 8). In these occurrences, it was evident that limited opportunity existed for social engagement and interaction, although this may be suited and cater towards people with developmental needs.

## 4. Discussion

The findings of this study overlapped significantly with previous social circus research [3–18]. Findings common across this and previous studies include the extensive range of physical, mental, cognitive, affective, and social benefits attained through participation, as well as experiences offered for joy, fun, friendship, inclusion, teamwork, trust, creativity, and personal growth.

Using the MOHO also provided new insights that had not been previously explicated in the social circus literature. The key aspect to emerge from analysis is the diversity within the circus. Whilst diversity was noted in previous literature as a difference between contemporary circus and social circus, diversity within the social circus environment, flexibility in times, and breadth of competence and components were highly apparent in the present study. High levels of flexibility, accessibility, and adaptability of the art form offered people with disabilities unique opportunities, not typically provided through other arts, physical, leisure, or recreation activities. Diversity was also exemplified by the fact that social circus was not always social, which could be seen enhancing participation and engagement for people who might not have otherwise chosen to engage.

Another aspect to emerge is the degree to which social circus accommodates differences in capacities, allowing opportunities for people of all ages and capabilities to develop and attain a wide range of skills, both circus-specific and generalisable to everyday life. Through attainment and refinement of circus-specific and everyday life skills, people with disabilities could experience autonomy and dignity of risk in a highly adaptable environment. Participants could also develop patience and respect for others, meet new people from a variety of backgrounds, and develop friendships outside the circus environment, which was noted to enhance occupational participation and engagement in life outside of circus.

Social circus also allowed people with disability to shape new identities, as the social circus sphere provides a safe, inclusive environment for people of all abilities to express themselves through a range of communicative forms, embrace various roles, and overcome boundaries and challenges. However, of particular significance, such opportunities were only noted because opportunities for active participation existed within the circus sphere. For people with disabilities, this is significant as people with disabilities often face a range of barriers and limitations to participation.

Occupational therapists have an imperative to improve people with disabilities' participation. “Participation” is arguably the overarching objective of the profession [38–40]. When considering children and young people with disabilities, opportunities to enhance participation are required, as participation is an essential element for development. It is through participation that children living with disabilities can increase emotional and social well-being, maintain or improve physical health, develop skills, and achieve enjoyment [41, 42].

Increasing opportunities for socialisation, engagement, and participation in meaningful occupations, such as leisure and recreation for people with disability of all ages, should also be addressed. Participation and engagement in leisure and recreational activities is a fundamental human right. International conventions and declarations, including the United Nations Convention on the Rights of the Child [43], as well as the Convention on the Rights of Persons with Disabilities [44], acknowledge:

“People with disability have the same right to take part in cultural life as other people do … to make it possible for people with disability to develop and use their creative, artistic and intellectual abilities, not only for their own benefit but for the benefit of society [44].”

It is acknowledged that for society to be truly inclusive, opportunities for socialisation in a range of community-based social settings are essential [44]. Given the identified participation potential, occupational benefits, experiences, inclusivity, and transference of circus skills to everyday tasks and occupations, social circus has promise for occupational therapy practice.

### 4.1. Strengths and Limitations

While it is acknowledged that limitations exist due to the emerging nature of theoretical video analysis, analysis of YouTube videos provided a unique opportunity for researchers to explore the current practice in a range of countries for people of all ages and abilities. The broad corpus of videos available on YouTube strengthened the research, ensuring varied perspectives and views. Inclusion of videos in languages other than English posed some limitations concerning transcription and omitted dialogue, yet visuals and subtitles provided for the consideration of social circus across various countries. The coding process allowed for videos to be coded, rewatched, and transcribed which permitted each relevant aspect (i.e., video audio, visuals, textual visuals, YouTube transcripts, and YouTube titles) to be reported.

The study included wide range of disabilities and conditions explicitly acknowledged within the videos, providing a rich description of the population, as it has typically remained omitted from the majority of prior research within this field. However, given the diversity of the sample, a degree of caution may be required when transferring the results to the broader population.

YouTube videos are inherently biased towards a positive or curated view of human experience, depending on the bias of the videographer capturing and narrating the experience. This is reflected in the present study through the predominance of positive themes. Few categories of themes reflected potential challenges or negative aspects of social circus (e.g., cost).

### 4.2. Implications for Social Circus

Through utilising the lens of the MOHO, this study highlights the importance of the physical, social, and occupational environments of the social circus setting. It is identified that when implementing social circus programs in a community setting, social circus practitioners should identify strategies to promote and enhance participation in social circus for people with all abilities. While social circus is highly adaptable at its core, additional strategies to promote and enhance participation and inclusion should be considered. Such strategies may include the adaptation of social circus programs (i.e., to include person-centred approaches), the inclusion of participants' significant others where required (i.e., options to include participant support networks in the delivery of social circus programs or classes), consideration and/or adaptation of areas of access and facilities, and consideration of suitable transportation options.

### 4.3. Implications for Occupational Therapists

Social circus can be implemented both as occupational therapy and in collaboration with occupational therapists. Social circus may be an appropriate means for achieving a wide range of occupational outcomes for people with and without disability across a diverse range of settings [3, 7].

The present study highlights the occupation potential of social circus when implemented as a therapeutic tool within contemporary practice. When and how to implement social circus in the practice context should be considered, as implementation should reflect consumers' personal interests, needs, and goals. Social circus as a form of therapeutic recreation would likely be of benefit to consumers including people interested in actively participating in the performing arts, individuals looking to learn new and transferable skills by means of a fun and inclusive occupation, people wanting to get out of their comfort zone and overcome boundaries and challenges, and those simply wanting to meet new people and increase their participation in physical activity in a community context. This may be due to the flexibility, adaptability, and inclusivity of the art form.

Social circus may provide an unrestricted platform for pursuing occupational performance goals when implemented as a therapeutic tool in the practice context. The creative platform allows for tailored and considered intervention, adaptation, and acquisition or development of skills (both circus-specific and generalisable to everyday life). Through social circus, attainment and refinement of a wide variety of goals and therapy outcomes may be achievable.

### 4.4. Implications for Researchers

This study focused on the benefits of social circus due to the inherently positive bias of YouTube videos. Future research into the feasibility of social circus may require alternate methodologies, such as interviews with social circus facilitators, participants, or families, to elucidate the potential challenges and pitfalls of social circus. This could support more informed decision-making regarding where and with whom social circus might be most appropriate.

For future research concerning social circus, it is suggested that researchers move to deductive methods such as randomised control trials which may provide a more rigorous way of determining whether a cause-effect relation exists between social circus intervention and depicted outcomes when implemented as a therapeutic tool within occupational therapy practice. Future research would be strengthened through the use of valid and reliable quantitative outcome measures that broadly capture the potential benefits noted in the qualitative literature.

Should researchers be interested in conducting research in the field of theoretical video analysis, the methods used in this paper allowed themes to be accurately identified and considered and new insights in the field to be developed. It is suggested that conducting studies utilising preestablished models or evidence-based conceptual frameworks provides scope for research questions to be accurately described and depicted. When not implementing such tools, it is suggested that researchers aim to identify specific and considered questions due to the involved and time-consuming nature of theoretical video analysis.

## 5. Conclusions

Based on the findings of this study, people with disabilities experience extensive occupational benefits from participation and engagement in social circus. While benefits identified in this study are consistent with the findings presented within both the grey and published literature, the identification and consideration of social circus through an occupational therapy theoretical lens offered a unique opportunity for further identification and consideration of the broad range of experiences offered through participation.

This study supports the use of social circus collaboration and implementation as a therapeutic tool within contemporary occupational therapy practice given extensive opportunities exist for enablement, adaptation, and participation. Through analysis, it was identified that social circus offers unique experiences for people with disabilities that may not be offered through typical art, physical, leisure, or recreation activities. Further research remains warranted to assess and evaluate its effectiveness in the practice context as an evidence-based intervention.

As Bolton says, “The evidence is overwhelming that in circus we have a medium and a message crying out to be heard and used by those with the energy, fearlessness, ingenuity, charm and the desire to come together and express themselves” (p.35 of [45]).

## Figures and Tables

**Table 1:** Seven stages of theoretical video analysis.

Stages
(1) An initial viewing of the videos
(2) Identify and adopt a theoretical framework (e.g., the MOHO)
(3) Rewatch the videos, interpreting and coding each video through the theoretical lens
(4) Review of the initial coding (e.g., by coresearcher/s)
(5) Consolidation of themes and categories
(6) Review of themes and categories (e.g., by coresearcher/s)
(7) An overall interpretation of the data through the theoretical lens as well as an examination of the suitability of the theoretical lens to examine the data

## Data Availability

Publicly available YouTube data were used to support this study, and video links are available in S1. These videos are cited at relevant places within the text as image references.

## Supplementary Materials

Supplementary Materials The selected YouTube video links have been presented as Supplementary Materials (S1).
