# Supplementary material for: Social Circus for People with Disabilities: A Video Analysis through the Lens of the MOHO
Source: Occup Ther Int. 2021 Mar 9;2021:6628482. doi: 10.1155/2021/6628482 (PMC7969102; doi:10.1155/2021/6628482)
Supplement: Supplementary Materials — The selected YouTube video links have been presented as Supplementary Materials (S1). [file 6628482.f1.pdf]

# Supplementary Materials

## S1: YouTube Links

https://www.youtube.com/watch?v=ylIDE0Fxqjg

https://www.youtube.com/watch?v=yvA6TLnEKBA

https://www.youtube.com/watch?v=Iz42IcaJFps

https://www.youtube.com/watch?v=aJVeH8GKzzk

https://www.youtube.com/watch?v=UvhLUkv9ox4

https://www.youtube.com/watch?v=qOKyaZKsrhQ

https://www.youtube.com/watch?v=KCjZxVPzcGs

https://www.youtube.com/watch?v=Cbg5jbFXE6k

https://www.youtube.com/watch?v=QRnyN78vKxw

https://www.youtube.com/watch?v=5KtaN__mQ38

https://www.youtube.com/watch?v=XQ_wSifZH-Q

https://www.youtube.com/watch?v=Yf_bbgjtubQ

https://www.youtube.com/watch?v=bGG0nO_LlZ4

https://www.youtube.com/watch?v=kz2ijT8jm4M

https://www.youtube.com/watch?v=L9LpwSP6cMo

https://www.youtube.com/watch?v=q7VerC4LxRs

https://www.youtube.com/watch?v=rUBfvAYgxxY

https://www.youtube.com/watch?v=kNdQ5L-MInE

https://www.youtube.com/watch?v=Z7CAdx1ZmSQ

https://www.youtube.com/watch?v=SdbAiTispNw

https://www.youtube.com/watch?v=eWlglurHF4A

https://www.youtube.com/watch?v=rBkREn6fzQ0

https://www.youtube.com/watch?v=db7XXiVBaSM

https://www.youtube.com/watch?v=l7AxyAH3Gls

https://www.youtube.com/watch?v=RQRe-eDrDa4

https://www.youtube.com/watch?v=JBTm8VhgUP0
